# Gastrointestinal Stromal Tumors Mimicking Gynecologic Disease: Clinicopathological Analysis of 20 Cases

**DOI:** 10.3390/diagnostics12071563

**Published:** 2022-06-27

**Authors:** Ying Liu, Maryam Shahi, Karin Miller, Christian F. Meyer, Chien-Fu Hung, T.-C. Wu, Russell Vang, Deyin Xing

**Affiliations:** 1 Department of Pathology, The Johns Hopkins Medical Institutions, Baltimore, MD 21231, USA; yliu341@jhmi.edu (Y.L.); shahi.maryam@mayo.edu (M.S.); kmill119@jhmi.edu (K.M.); chung2@jhmi.edu (C.-F.H.); wutc@jhmi.edu (T.-C.W.); rvang1@jhmi.edu (R.V.); 2 Department of Laboratory Medicine and Pathology, Mayo Clinic, Rochester, MN 55901, USA; 3 Department of Oncology, The Johns Hopkins Medical Institutions, Baltimore, MD 21231, USA; cmeyer13@jhmi.edu; 4 Department of Gynecology and Obstetrics, The Johns Hopkins Medical Institutions, Baltimore, MD 21231, USA

**Keywords:** gastrointestinal stromal tumor, pelvic mass, *c-KIT*, *RB1*, *FGFR3*

## Abstract

Diagnosis of pelvic gastrointestinal stromal tumors (GISTs) can be challenging because of their nonspecific presentation and similarity to gynecological neoplasms. In this series, we describe the clinicopathological features of 20 GIST cases: 18 patients presented with pelvic mass and/or abdominal pain concerning gynecological disease; 2 patients presented with a posterior rectovaginal mass or an anorectal mass. Total abdominal hysterectomy and/or salpingo-oophorectomy (unilateral or bilateral) were performed in 13 cases. Gross and histological examination revealed that the ovary/ovaries were involved in three cases, the uterus in two cases, the vagina in two cases and the broad ligament in one case. Immunohistochemically, all tumors (20/20, 100%) were diffusely immunoreactive for c-KIT. The tumor cells were also diffusely positive for DOG-1 (10/10, 100%) and displayed focal to diffuse positivity for CD34 (11/12, 92%). Desmin was focally and weakly expressed in 1 of the 14 tested tumors (1/14, 7%), whereas 2 of 8 tumors (2/8, 25%) showed focal SMA positivity. At the molecular level, 7 of 8 (87.5%) GISTs with molecular analysis contained *c-KIT* mutations with the second and third *c-KIT* mutations detected in some recurrent tumors. In addition to *c-KIT* mutation, a pathogenic *RB1* mutation was detected in two cases. We extensively discussed these cases focusing on their differential diagnosis described by the submitting pathologists during consultation. Our study emphasizes the importance of precision diagnosis of GISTs. Alertness to this entity in unusual locations, in combination with clinical history, morphological features as well as immunophenotype, is crucial in leading to a definitive classification.

## 1. Introduction

Gastrointestinal stromal tumors (GISTs) are rare tumors with an estimated yearly incidence of 10–15 per million [1,2]. Although accounting for less than 1% of all gastrointestinal tumors, GISTs represent approximately 80% of mesenchymal tumors of the gastrointestinal tract, commonly occurring in the stomach (56%), followed by the small bowel (32%) and other sites including the colon, rectum, esophagus and extra-gastrointestinal locations [3]. These tumors are thought to arise from specialized pacemaker cells called interstitial cells of Cajal [4] and display a variety of morphologies (spindle, epithelioid, or mixed histology) [5,6]. Immunohistochemically, more than 90% of tumors are immunoreactive for c-Kit and DOG-1 and 50–70% for CD34, serving as diagnostic markers [7]. At the molecular level, 60–75% of GIST cases harbor pathogenic driver mutations in the *c-KIT* gene and 10–15% have *PDGFRA* mutations [8,9,10]. Other oncogenic drivers, including mutations in the genes of subunits of the enzyme succinate dehydrogenase, *BRAF*, *RAS*, and *NF1* genes, have been reported in the cases without *c-KIT* and *PDGFRA* mutation [11,12,13,14].

The clinical presentation of a GIST is variable and depends on the location, tumor size and growth pattern [15]. In many patients, GISTs are often an incidental finding during operation and/or detected through screening examinations with unspecific symptoms. When presented as a pelvic mass, diagnosis of a GIST could be challenging due to its nonspecific presentation and similarity in appearance to gynecological neoplasms [16,17,18,19,20,21,22,23]. On rare occasions, these tumors can present as a rectovaginal mass for which the differential diagnosis is broad [24,25]. In this study, we describe the clinicopathological features of 20 GIST cases presenting as a pelvic mass (18 cases including one with vaginal involvement), a posterior vaginal mass (one case), or an anorectal mass (one case), all of which clinically mimic gynecologic disease. We also describe molecular alterations in eight cases.

## 2. Materials and Methods

### 2.1. Case Selection

38 GIST cases were identified in the files of The Johns Hopkins Hospital between January 2000 and January 2022 through searching “gastrointestinal stromal tumor” and “pelvic mass”, “rectovaginal mass”, or “anorectal mass”. Twenty of the cases with available H&E and immunohistochemical slides, including 11 cases from in-house service, and the remaining 9 cases from consultation files, were retrieved. Histologic features of these cases were reviewed by 2 board-certified pathologists (Y.L. and D.X.) and clinicopathological features, including clinical presentation, age at diagnosis, operative procedure, tumor site and size, and follow-up information, were analyzed. The study was approved by the Institutional Review Board at the Johns Hopkins Hospital.

### 2.2. Immunohistochemistry

Immunohistochemical staining was performed in the Johns Hopkins Immunopathology Laboratory on formalin-fixed, paraffin-embedded tissue sections, using Ventana Benchmark automation and the Ultra View detection kit (Ventana Medical Systems, Tucson, AZ, USA) as previously described [26,27]. All immunostains were performed at the time of diagnosis for primary or recurrent tumor. Antibodies from Cellmarque, Hot Springs, AZ, included: c-Kit (YR145), DOG-1 (SP31), CAM5.2 (B22.1&B23), Calretinin (Polyclonal), Cyclin D1 (SP4); antibodies from Ventana, Tucson, AZ, USA, included: CD34 (QBE/10), CD68 (KP-1), SMA (1A4), AE1/AE3 (pck-26), Actin (MOUSE), S100 (4C4.9), ER (SP-1), Melan A (A103); antibodies from DAKO, Carpinteria, CA, USA, included Desmin (D33) and Caldesmon (h-CD); antibodies from Abcam, Cambridge, MA, included ALK (5A4), Cathepsin K (3F9), and SDHB (21A11AE7); antibodies from Leica, Bannock Burn, IL, USA, included CD10 (MS/56C6) and PR (1E2); the rest of the antibodies included SOX-10 (N-20, Biocare, Concord, CA, USA), HMB-45 (HMB45, Novocastra, Newcastle upon Tyne, UK), Inhibin (R1, Serotec, Raleigh, NC), STAT6 (D-1, Santa Cruz, Santa Cruz, CA, USA), and SF-1 (n1665, Invitrogen, Waltham, MA, USA).

### 2.3. DNA Extraction

Formalin-fixed paraffin-embedded (FFPE) tumor and corresponding normal tissues were identified by H&E staining and subsequently were macrodissected (with tumor elements accounting for about 60% or more of the section area). Genomic DNA was extracted using a QIAamp DNA FFPE Tissue Kit with an adopted protocol (Qiagen, Valencia, CA, USA). Briefly, slides bearing paraffin embedded tissue were baked at 68 °C for 20 to 30 s; the tissue was deparaffinized 3 times with xylene, and residual xylene was removed by washing through serial dilutions of ethanol. The rest of the procedure followed the manufacturer’s instruction.

### 2.4. Molecular Analysis

*c-KIT* mutation was analyzed by PCR [cases 3, 6 (primary tumor), 7] or in-house next-generation sequencing (NGS) service [cases 1, 6 (recurrent tumor), 11–13, 18] at the time of initial diagnosis or recurrence. Briefly, for PCR-based analysis, DNA was extracted from the tissue and subject to PCR amplification using primers to exons 9, 11, 13, 17 and 18 of the *c-KIT* gene. The amplification products were analyzed by bi-directional direct DNA Sanger sequencing using capillary gel electrophoresis and fluorescence detection. For NGS-based analysis, DNA was either extracted from tissue and captured with Kapa Roche reagents and Integrated DNA Technology (IDT) probes, and sequenced using Illumina paired end technology [26,28] or extracted from the tissue and subjected to multiplex PCR amplification using the Ion AmpliSeq Cancer HotSpot Panel v2 (Life Technologies) and sequenced using ion semi-conductor sequencing technology on the Ion Torrent S5 XL.

*FGFR3* c.433G > A (p.G145S) mutation/variant was further assessed by Sanger sequencing. Briefly, 50 ng of DNA was amplified by PCR with *Taq* DNA Polymerase and Standard *Taq* Buffer (New England BioLabs, MA). The following primers were used for amplification: 1F: 5′-CAGGAAGTGCTGCCCAAATG-3′, 1R: 5′-CCTCAGCTGCCTGTGAAGGG-3′, and 2F: 5′-GCAGGTTGGGCATTGGTTGC-3′. The reaction for amplification of first-round PCR primers (1F/1R) was carried out in the following conditions: an initial melting step of 2 min at 95 °C, followed by 32 cycles of 30 s at 94 °C, 30 s at 51 °C and 30 s at 72 °C, and a final elongation of 7 min at 72 °C. The PCR products amplified by 1F/1R primers were diluted 10 times and used as templates of a second PCR amplification (nested PCR) with another pair of primers 2F/1R that was carried out in similar reaction conditions except for an annealing temperature of 56 °C. DNA sequencing of the purified DNA products was performed using the ABI 3730 High-Throughput DNA Sequencer. The mutations and variations were analyzed using Unipro UGENE software.

## 3. Results

### 3.1. Clinicopathological Features

Clinicopathological features are provided in Table 1. In this series of 20 cases, the patients ranged in age from 23 to 83 years (mean, 51.7; median, 51.5) at the initial diagnosis. Eighteen patients presented with a pelvic mass and/or abdominal pain concerning for gynecological disease, of which, three patients had a history of GISTs (two stomach, one with unavailable primary site information). One patient presented with a posterior vaginal mass between the vagina and rectum and another patient presented with an anorectal mass. Given the possibility of gynecologic lesion or involvement, total abdominal hysterectomy and/or salpingo-oophorectomy (unilateral or bilateral) were performed in 13 cases.

**Figure 1:**
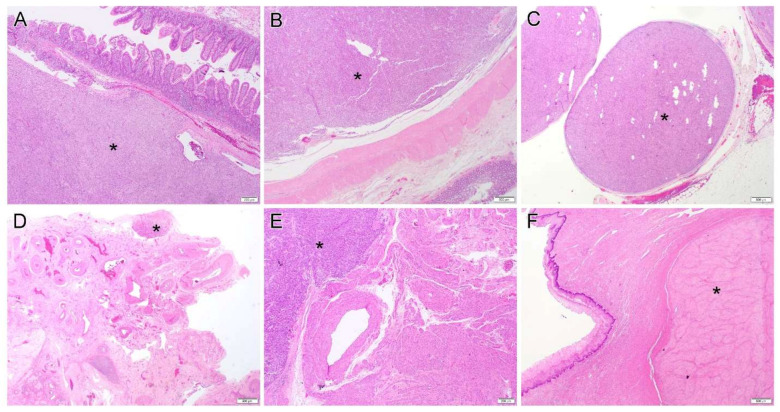
(**A**–**C**). Gastrointestinal stromal tumor originates/involves small bowel (**A**, case 4), colon (**B**, case 2) and omentum (**C**, case 13). (**D**–**F**). Representative pictures of gynecologic organ involvement: periovarian tissue (**D**, case 4), uterus (**E**, case 17) and vagina (**F**, case 15). Star (*) indicates the tumor. Original magnification: (**A**,**E**), ×40; (**B**–**D**,**F**), ×20.

Analysis of the clinical information including history and gross/histologic examination indicated likely small bowel origin in 13 cases, rectum in 3 cases, stomach in 3 cases and 1 recurrent pelvic GIST with unavailable primary site information. Although the tumors ranged in size from 2.7 to 27 cm (mean, 12.7; median, 11), 12 cases exhibited one or two main tumors associated with multiple smaller nodules that were disseminated in the peritoneal/pelvic cavity (Figure 1A–C). In terms of gynecologic organs, the ovary/ovaries were involved in three cases (Figure 1D), the uterus in two cases (Figure 1E), the vagina in two cases (Figure 1F), and the broad ligament in one case. Two patients with a history of a gastric GIST showed multiple peritoneal organ involvement and liver metastasis.

Follow-up information was available in 15 cases. At the last follow-up, five patients showed no evidence of residual disease with a median follow-up time of 17 months. Four patients died: two died of disease at 30 months and 108 months, respectively; one patient died of acute myeloid leukemia; and one patient died with from an unknown reason at 84 months. Six patients were still alive (median follow-up 120 months) but had multiple recurrence or disease progression.

### 3.2. Morphology and Immunohistochemical Findings

**Figure 2:**
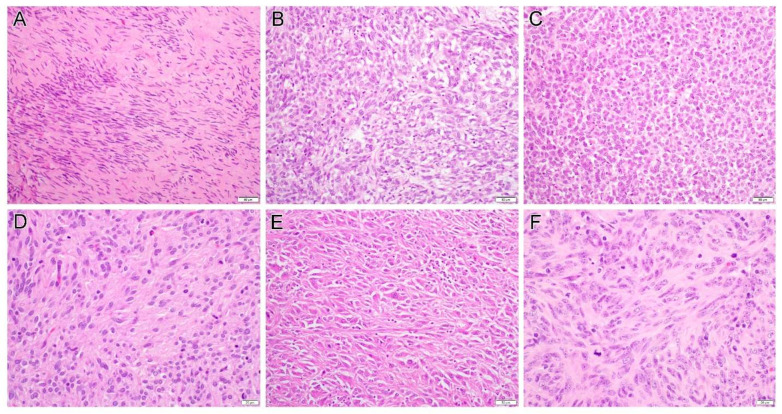
Examples of gastrointestinal stromal tumor histology include sclerosing spindle cell (**A**, case 15), vacuolated (**B**, case 2) or epithelioid (**C**, case 2) morphology. Some tumors focally exhibited neuropil-like (**D**, case 10), sarcomatoid appearance (**E**, case 5) or significant cytologic atypia and readily recognizable mitoses (**F**, case 13). Original magnification: (**A**–**C**,**E**), ×200; (**D**,**F**), ×400.

**Figure 3:**
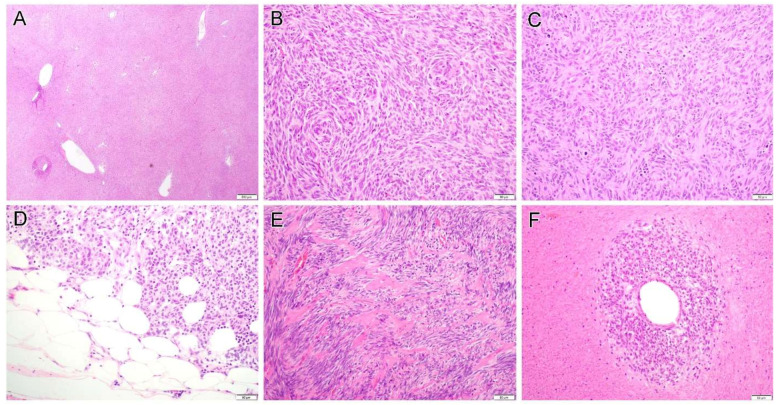
Gastrointestinal stromal tumor displays a variety of growth pattern including hemangiopericytoma-like pattern (**A**, case 9), storiform (**B**, case 9) and haphazard (**C**, case 10) tumor growth, and fat involvement (**D**, case 13) can be seen in some areas. Some tumors contained hyalinized collagens (**E**, case 11). In some cases, viable cells are present only around the blood vessel and ghost-like outlines of necrotic atypical tumor cells can still be discerned in the surrounding tissue (**F**, case 16). Original magnification: (**A**), ×20; (**B**–**F**), ×200.

Morphological features and immunohistochemical findings are summarized in Table 2. Cases 1 to 13 were of tumors of likely small bowel origin which displayed either pure spindle (seven cases) or mixed spindle and epithelioid features (six cases). The detailed histology included sclerosing spindle cell (Figure 2A), vacuolated (Figure 2B) or epithelioid (Figure 2C) morphology. Some tumors focally exhibited neuropil-like (Figure 2D) or sarcomatoid appearance (Figure 2E). While some tumors were low-grade, others displayed significant cytologic atypia and readily recognizable mitoses (Figure 2F). Hemangiopericytoma-like pattern (Figure 3A), storiform (Figure 3B) and haphazard (Figure 3C) tumor growth, and fat involvement (Figure 3D), can be seen in some areas. Although some tumors contained hyalinized collagens (Figure 3E), overt evidence of skeinoid fibers, a feature described in intestinal GIST, was not seen. Of these 13 cases, the mitotic rate varied from 2 to 40 per 50 high power fields (HPFs; median, 8), and tumor necrosis was present in seven cases. Interestingly, in some areas, viable cells were present only around the blood vessel and ghost-like outlines of necrotic atypical tumor cells can still be discerned in the surrounding tissue (Figure 3F).

**Figure 4:**
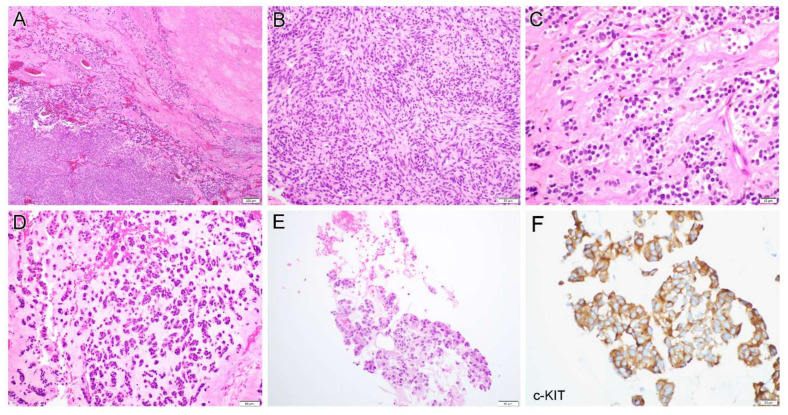

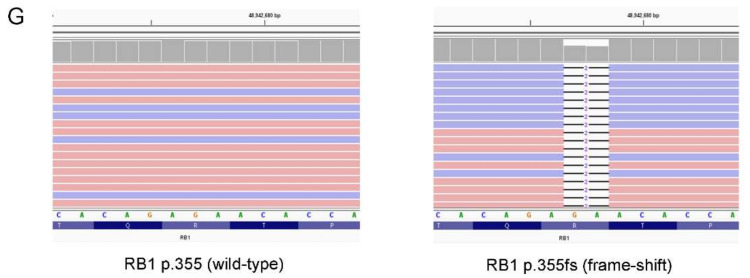
Case 6 was a 32-year-old woman with gastrointestinal stromal tumor (GIST) that displayed a sheet-like growth pattern with extensive hyalinization (**A**), composed of spindle cells (**B**) and uniform epithelioid cells (**C**) with the former arranged in a pattern analogous to Verocay bodies. Myxoid changes (**D**) were present in some areas. The most recent biopsy (108 months after the initial diagnosis) showed a recurrent GIST with epithelioid morphology (**E**) with diffuse c-KIT expression (**F**). Next-generation sequencing revealed a pathogenic *RB1* p.R355fs mutation (**G**, right) in the most recent biopsy specimen but not in the primary (**G**, left) or previous recurrent tumors. Original magnification: (**A**), ×40; (**B**,**D**,**E**), ×200; (**C**,**F**), ×400.

Case 6 was a 32-year-old woman who developed abdominal pain and presented with a pelvic mass. A biopsy revealed a GIST with mixed spindle and epithelioid features and a subsequent tumor debulking procedure was performed. Gross examination revealed multiple mass lesions (largest 2.7 cm) involving the jejunum (primary site), mesentery, bladder, appendix and bilateral ovaries. Histologically, the tumor displayed a sheet-like growth pattern with extensive hyalinization (Figure 4A). The tumor was composed of spindle cells (Figure 4B) and uniform epithelioid cells (Figure 4C) with the former arranged in a pattern analogous to Verocay bodies (Figure 4B). Myxoid changes (Figure 4D) were present in some areas. The lesion underwent multiple recurrences, and the most recent biopsy (108 months) showed a recurrent GIST with epithelioid morphology (Figure 4E).

Two spindle cell type GISTs and one epithelioid GIST were of probable rectal origin (cases 14–16). The mitotic count was 3, 3, and 53 per 50 HPFs, respectively. Three GISTs (cases 17–19) were of gastric origin, of which, two had known primary site history. The third patient presented with pelvic mass and had multiple tumor nodules in the abdominal cavity with the main tumor in the stomach. The two high-grade tumors displayed epithelioid or mixed morphology with a mitotic rate of 33 and 27 per 50 HPFs, respectively.

**Figure 5:**
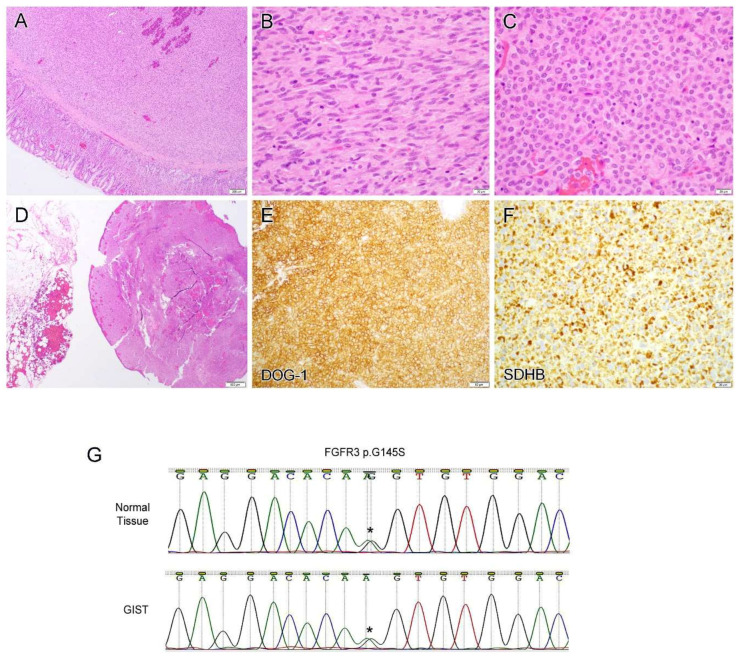
Case 18 was a 23-year-old woman with gastric-type gastrointestinal stromal tumor (**A**) which displayed spindle (**B**) and epithelioid morphology (**C**) and omental involvement (**D**). The tumor cells diffusely expressed DOG-1 (**E**) and displayed retained SDHB staining (**F**). Next-generation sequencing and Sanger sequencing revealed a *FGFR3* p.G145S mutation/variant which was detected in both normal tissue and tumor (**G**). Star (*) indicates the mutation/variant site. Original magnification: (**A**), ×40; (**B**,**C**), ×400; (**D**), ×20; (**E**,**F**), ×200.

Case 18 was a 23-year-old woman with sudden onset of abdominal pain. An emergency laparoscopic procedure revealed multiple mass lesions involving the omentum and pelvic cul-de-sac with pathology-confirmed GIST. A subsequent partial gastrectomy and omentectomy demonstrated a gastric GIST (Figure 5A) with spindle (Figure 5B) and epithelioid morphology (Figure 5C) and omental involvement (Figure 5D). The tumor cells diffusely expressed DOG-1 (Figure 5E) and displayed retained SDHB staining (Figure 5F).

All tumors (20/20, 100%) were diffusely immunoreactive for c-KIT (Figure 4F). The tumor cells were also diffusely positive for DOG-1 (10/10, 100%, Figure 5E) and displayed focal to diffuse positivity for CD34 (11/12, 92%). Desmin was focally and weakly expressed in one of fourteen tested tumors (1/14, 7%), whereas two of eight tumors (2/8, 25%) showed focal SMA positivity. All tumors were negative for AE1/AE3, Inhibin, SF-1 and S100, if these markers were tested.

### 3.3. Molecular Findings

Molecular genetic studies were performed on eight cases. *c-KIT* exon 11 deletion/insertion mutation and c.1510insGCCTAT (p.504inAlaTyr) exon 9 insertion mutation were detected by PCR-based mutation analysis in cases 3 and 7, respectively. NGS-based analysis revealed a variety of *c-KIT* mutations including A502_Y503 (insertion, cases 1 and 13), p.W557R (missense, case 11) and p.M552_V555del (deletion, case 12). In case 1, a pathogenic *RB1* splicing site mutation c.1128-1G > C was also detected. In case 13, a second *c-KIT* mutation p.N822K was detected in the current specimen but not in the previous one, indicating a tumor progression with imatinib resistance and an interval change (43 months). Likewise, in case 6, a *c-KIT* p.Y578_D579dup Exon 11 insertion mutation was detected by PCR in the primary tumor. While the same mutation was also present in the recurrent tumors, additional *c-KIT* mutations pV654A (exon 13) and L783V (exon 16) were detected. In the most recent specimen, an *RB1* mutation p.R355fs (Figure 4G) was present, in addition to three *c-KIT* mutations; this mutation was not detected in the primary or previous recurrent tumors. Case 18 contained an *FGFR3* p.G145S mutation/variant detected by NGS analysis. A confirmative Sanger sequencing revealed this was a germline variant which was detected in both normal tissue and the tumor (Figure 5G).

## 4. Discussion

From a pathology standpoint, only a small number of pelvic GISTs mimicking gynecologic disease have been documented in the literature [16,17,18,19,20,21,22,23,24,25,29,30]. In this study, we described 20 cases with emphasis on their clinicopathological features, immunohistochemical findings, and in some cases, their molecular alterations. In fact, a GIST presenting as a pelvic mass usually poses diagnostic challenges, particularly in a biopsy specimen.

Of the nine consult cases, a smooth muscle tumor was considered by the contributing pathologists as the top differential diagnosis in two cases. Case 5 was a 53-year-old woman who had multiple mass lesions involving the small bowel, peritoneum, omentum and bilateral ovaries. The tumor displayed spindle and epithelioid morphology and sarcomatoid features with a frozen diagnosis of leiomyosarcoma. Another patient (case 10) also presented with multiple peritoneal masses that were considered as smooth muscle lesion, favor malignant. The tumors in both cases showed focal and weak SMA staining, were negative for desmin and diffusely positive for c-KIT. This immunoprofile supports interpretation as a GIST rather than a smooth muscle tumor. Of note, focal positivity of caldesmon was observed in case 10. Caution should be taken when interpreting positive caldesmon in GISTs since this marker can be expressed in up to 80% of GIST cases [5].

In this series, 12 cases exhibited multiple mass lesions that were disseminated in the peritoneal/pelvic cavity, a distribution pattern similar to disseminated peritoneal leiomyomatosis (DPL). DPL is characterized by multiple smooth-surfaced and variably sized nodules or masses scattered over the peritoneum and in the omentum [31]. These lesions are generally small (<1 cm), consisting of histologically bland smooth-muscle cells with no or low mitotic count. In contrast, pelvic GISTs, either primary or metastasized from gastric origin, usually display one or two main tumors associated with multiple smaller nodules. Consistently, the median size of the main tumors in this series is 11 cm (ranging 2.7 to 27 cm). Immunohistochemically, similarly to other smooth muscle tumors, DPL is typically positive for desmin that is rarely expressed in GIST [19,32]. A panel of immunohistochemical markers including c-KIT and DOG-1 can facilitate the diagnosis.

Another differential diagnosis of the pelvic GIST is an endometrial stromal neoplasm, especially a low-grade endometrial stromal sarcoma (LGESS), which was the top consideration by the outside pathologist for case 18. LGESS is composed of cells resembling stromal cells of proliferative phase endometrium [33]. The tumor cells are typically small with scant cytoplasm and uniform, oval to fusiform nuclei. Delicate small arterioles, hyaline plaques and foamy histiocytes can be seen. Primary ovarian or pelvic endometrioid stromal sarcomas are usually associated with endometriosis. The diagnosis of metastatic LGESS is related to a primary tumor in the uterus. Interestingly, cases 2 and 17 showed uterus involvement by GIST. Without any other information, this observation alluded to a smooth muscle tumor or LGESS, which are the most common mesenchymal tumors in the uterus. Unlike the GIST, the LGESS is typically immunoreactive for CD10 but negative for c-KIT and DOG-1.

Other mesenchymal tumors in the differential diagnosis described by the contributing pathologists include hemangiopericytoma (case 14), solitary fibrous tumor (SFT, case 14) and perivascular epithelioid cell tumor (PEComa, case 20). In fact, some GISTs display a hemangiopericytoma-like growth pattern, and similar to GIST, both hemangiopericytoma and SFT can express CD34. A PEComa is characterized by a variable admixture of epithelioid and spindled cells with the former arranged in nested or diffuse patterns and the latter arranged in short fascicles and nests [34]. In fact, this morphology can be encountered in a GIST. Immunohistochemically, STAT6 is a sensitive and specific marker for SFT, and HMB-45, Melan A, and cathepsin K can be used to facilitate a diagnosis of PEComa.

In case 9, the favored diagnosis by the contributing pathologist was an adult granulosa cell tumor (AGCT). The AGCT tumor cells usually display epithelioid morphology with scant pale cytoplasm and uniform, round to oval nucleoli [35]. Nuclear grooves are a characteristic feature of this tumor but might not be conspicuous. In some tumors, the cells are spindled and/or contain a variable amount of fibromatous or thecomatous stroma. These features resemble a mixed spindle cell and epithelioid type GIST. In this series, 18 of 20 cases (90%) presented with a pelvic mass and/or abdominal pain and only three cases had a known history of GIST. Not surprisingly, these initial clinical manifestations can lead to a strong suspicion of ovarian neoplasms. In one study, five cases of GIST metastatic to the ovary were reported, and most of the ovarian tumors in that series were initially diagnosed as tumors of other types than GIST [19]. Normally, since pelvic GISTs commonly manifest as multiple mass lesions that require a tumor-debulking procedure, an initial evaluation often includes this entity as a differential diagnosis. However, in a biopsy or local excision specimen without information about any involvement of the gastrointestinal tract, diagnosis can be challenging. In this setting, some benign ovarian tumors such as fibroma/fibrothecoma also enter the differential diagnosis; SF-1 and inhibin, in addition to c-KIT and DOG-1, can be used to differentiate these lesions.

A GIST in the rectovaginal septum is extremely rare as we reported three cases in this series. Due to its unusual location and clinical presentation, the precise diagnosis is even more challenging compared with the cases in other locations. In addition to the entities discussed above, the differential diagnosis in this location also includes schwannoma, aggressive angiomyxoma, angiomyofibroblastoma, cellular fibroma and dermatofibrosarcoma protuberans if the lesion is close to the skin [24,25]. Consideration of GIST in the differential diagnosis of mesenchymal neoplasms in the rectovaginal septum with a panel of immunostainings can assist in the diagnosis.

The literature indicates that approximately 85% of GISTs harbor gain-of-function mutations of the *c-KIT* (75%) or *PDGFRA* (10%) oncogene as a tumorigenic driver [8,9,10]. In this series, seven of eight (87.5%) GISTs with molecular analysis contained *c-KIT* mutations. Interestingly, the second *c-KIT* mutation (cases 6 and 13) and the third one (case 6) were also detected in the recurrent tumor but not in the primary lesion, indicating disease progression. In addition to *c-KIT* mutation, a pathogenic *RB1* mutation was detected in two cases. In particular, molecular analysis was performed four times in case 6, including on the primary tumor and three recurrent ones at different time points, with a frameshift *RB1* mutation only detected in the most recent lesion. Consistent with our findings, one study showed that *TP53* and *RB1* mutations seem to be restricted to high-risk/malignant GISTs, although occurring at a relatively low frequency [36]. In another study, a whole exome sequencing-based analysis demonstrates that genomic events (mutation and amplification) targeting cell cycle-related genes, including *CDKN2A*, *RB1* and *TP53*, are associated with GIST progression to malignant disease [37]. Ideally, all cases need to be assessed by immunohistochemical markers c-KIT, DOG-1 and CD34, as well as molecular analysis. A limitation of this study is that our current series consisted of cases that were retrospectively diagnosed over a period of more than 20 years, some of which were consultation cases from outside the institution. For this reason, performing these ancillary analyses was not possible in all cases.

Case 18 was a unique case. The patient was a 23-year-old woman with sudden onset of abdominal pain and multiple omental and pelvic lesions. The biopsy specimen confirmed a diagnosis of GIST and subsequent partial-gastrectomy tumor-debulking demonstrated a lesion of gastric origin. She reported a similar symptom when she was 13-year-olds but nothing was found at that time except for hemoperitoneum. NGS analysis of her GIST lesion revealed a quadruple wild-type tumor (no mutations in *c-KIT*, *PDGFRA*, *SDH*, RAS-P [*RAS*, *BRAF*, *NF-1*]) with a *FGFR3* p.G145S mutation/variant which is of a germline rather than somatic variation. Since this variant has never been described previously, it is difficult to ascertain whether this is a pathogenic germline mutation or a single nucleotide polymorphism (SNP). On the other hand, FGF/FGFR pathway activation in GISTs, including gain-of-function mutations, oncogenic gene fusions, and overexpression, has been detected in quadruple wild-type, SDH-deficient, or *KIT*-mutant GISTs [38]. In particular, concomitant *FGFR3* mutations were observed in two GIST patients: one with c-*KIT* gene mutation [39] and another with *PIK3CA* and *JAK1* gene mutations [40]. An experimental study demonstrated that signaling crosstalk between c-KIT and FGFR3 activated the MAPK pathway to promote resistance to imatinib, providing a mechanistic rationale to target FGFR3 as a strategy to improve the treatment of GIST patients with de novo or acquired resistance to imatinib [41].

In summary, we described 20 GIST cases presenting as a pelvic mass, rectovaginal mass or anorectal mass and mimicking gynecologic disease, with emphasis on their differential diagnosis described by the submitting pathologists during consultation. Establishing the diagnosis of GIST has both prognostic and therapeutic implications, and alertness to this entity in unusual locations, in combination with clinical history, morphological features as well as immunophenotype, is crucial in leading to precision diagnosis.

## Figures and Tables

**Table 1:** Clinicopathological features.

Case	Age and the Year at the Original Diagnosis	Clinical Presentation	Surgical Procedure *	Site and Size of Primary Tumor	Gynecologic Organ Involvement	Patient Outcome
1	56 (2019)	Abdominal pain and pelvic mass	TAH, LSO, small bowel resection, omental biopsy	Ileum, 12.6 cm	Not involved	16 months with tumor debulking (multiple pelvic mass, largest 8.4 cm); 29 months with residual disease, on Imatinib
2	59 (2006)	Abdominal pain and omental/pelvic mass	TAH, LSO, small bowel and colon resection, OMT	Multiple mass lesions involving small bowel, colon, bladder, and pelvis; largest 21.3 cm	Uterus involved	Diagnosed with small bowel GIST at age 59 and managed conservatively with Imatinib. Debulking procedure was performed at age 72. Currently on palliation
3	62 (2006)	Pelvic mass	TAH, BSO, small bowel resection, liver segmental resection, CCY	Small bowel, 4.7 cm	Not involved	No residual GIST. The patient developed acute myeloid leukemia (ALL) at age 76. Died of ALL at age 77
4	80 (2009)	Abdominal pain and pelvic mass	TAH, BSO, small bowel resection, OMT, APPY	Multiple mass lesions involving small bowel, peritoneum and omentum; largest 14 cm	Left ovary involved by microscopic focus of metastatic GIST	Died at age 87
5	53 (2012)	Pelvic mass	TAH, BSO, small bowel and sigmoid colon resection, OMT	Multiple mass lesions involving small bowel, peritoneum and omentum; largest 20 cm	Bilateral ovaries involved by metastatic GIST	NA
6	32 (2013)	Pelvic mass	Tumor debulking, APPY, OMT	Multiple mass lesions involving jejunum, mesentery, bladder, appendix; largest 2.7 cm	Bilateral ovaries involved by metastatic GIST	Recurrent at the age 40. Currently (41 years old) on sunitinib
7	45 (2011)	Pelvic mass	Tumor debulking, small bowel resection, APPY, OMT, BSO	Multiple mass lesions involving small bowel, mesentery, rectum; largest 10 cm	Not involved	Recurrent at the age 49. Currently (56 years old) on palliation
8	44 (2019)	Right pelvic mass	TAH, bilateral ovarian cystectomy, tubal ligation, tumor excision	2 mass lesions (6.5 cm and 5.3 cm) involving ileum and broad ligament	Not involved	NA
9	51 (2020)	Pelvic mass	Excision and APPY	17 cm, attached to small bowel and sigmoid colon	Not involved	NA
10	83 (2011)	Pelvic mass	Small bowel resection, OMT, APPY	Multiple mass lesions involving small bowel, omentum, bladder peritoneum; largest 14.5 cm	Not involved (history of TAH and BSO for benign lesion)	30 months, died of disease
11	53 (2021)	Pelvic mass	Small bowel resection, omental biopsy, APPY, myomectomy, bilateral salpingectomy	22.5 cm solid and cystic mass arising from the wall of the small bowel; status post neoadjuvant treatment	Not involved	4 months, no evidence of disease
12	36 (2020)	Pelvic mass	TAH, BSO, OMT	27 cm mass focally attached to the proximal ileum	Not involved	14 months, no evidence of disease
13	33 (2016)	Pelvic mass	Small bowel resection, tumor debulking, APPY, CCY	27 cm small bowel mass with intraperitoneal spread	Not involved	52 months, no evidence of disease
14	47 (2017)	9 cm posterior vaginal mass, between rectum and vagina	Biopsy	Rectum; 9 cm by imaging	Vagina	NA
15	52 (2008)	Pelvic mass	Colectomy	Rectum; 6.4 cm	Posterior vagina	161 months, multiple recurrence, currently disease progression
16	70 (2012)	Anorectal mass	Rectum resection, TAH, BSO	Rectum; 12 cm	Not involved	17 months, no evidence of disease
17	48 (2008)	Pelvic mass; history of GIST	Partial gastrectomy, liver segmental resection, CCY, TAH, RSO	Stomach, 7 cm	Uterus involved by metastatic GIST	Gastric GIST with multiple recurrence involving liver, omentum, diaphragm, pelvic sidewall, uterus, sacrum, and rectus. Died of disease at age 57
18	23 (2018)	Abdominal pain and omental/pelvic mass	OMT, CCY, partial gastrectomy	Stomach origin; multiple mass lesions involving omentum and pelvic cul-de-sac; largest 3.5 cm	Not involved	36 months, no evidence of disease
19	41 (2005)	Pelvic mass; history of GIST	Partial gastrectomy, liver segmental resection, CCY, TAH, BSO	Stomach, 7 cm; liver metastasis; involving gallbladder, mesentery, pelvic sidewall	Not involved	76 months, multiple recurrence, last follow-up with disease progression
20	66 (2019)	Left pelvic mass; history of GIST	Excision	9 cm, unknown primary site	Unknown	NA

APPY: appendectomy; BSO: bilateral salpingo-oophorectomy; CCY: cholecystectomy; GIST: gastrointestinal stromal tumor; LSO: left salpingo-oophorectomy; OMT: omentectomy; RSO: right salpingo-oophorectomy; TAH: total abdominal hysterectomy. * Some procedures were performed at different time points.

**Table 2:** Morphology, immunohistochemistry, and molecular analysis.

Case	Tumor Morphology	Necrosis	MF/50 HPFs	c-KIT	DOG-1	CD34	Desmin	Other IHCs	Molecular Analysis
1	spindle	Present	36	+	+	ND	-	SMA-, SOX10-	c-KIT A502_Y503 (insertion); RB1 c.1128-1G > C
2	epithelioid and spindle	Present	8	+	+	+ (weak)	ND	AE1/AE3-	ND
3	spindle	Absent	17	+	ND	+	-	ND	c-KIT exon 11 deletion/insertion mutation
4	epithelioid and spindle	Present	35	+	ND	+	-	Actin+ (weak), S100-	ND
5	epithelioid and spindle	Absent	6	+	ND	-	-	Actin-, SMA+ (weak), Cam5.2-, S100-, HMB45-, Melan A-, Inhibin-, calretinin-	ND
6	epithelioid and spindle	Absent	7	+	+	ND	ND	SMA-	c-KIT Exon 11 mutation (1732_1737dup: p.Y578_D579dup), additional mutations pV654A (exon 13) and L783V (exon 16); RB1 p.R355fs
7	spindle	Absent	3	+	ND	+	ND	ND	c-KIT c.1510insGCCTAT(p.504inAlaTyr) exon 9 mutation
8	spindle	Present	2	+	+	+	-	AE1/AE3-, HMB45-, ER-, PR-, CD68-, S100-	ND
9	epithelioid and spindle	Present	20	+	+	ND	-	SF-1-, Inhibin-, HMB45-, Cathepsin K+ (weak)	ND
10	epithelioid and spindle	Present	40	+	ND	+	-	Caldesmon+ (focal), SMA+ (weak), Actin-, ER-, PR-, HMB45-, S100-	ND
11	spindle	Present	2	+	+	ND	-	S100-	c-KIT p.W557R
12	spindle	Absent	6	+	ND	+	ND	AE1/AE3-, Cam5.2-, Calretinin-, Synaptophysin-, SMA-	c-KIT p.M552_V555del
13	spindle	Absent	38	+	+	ND	ND	ND	c-KIT p.A502_Y503dup and p.N822K
14	spindle	Absent	3	+	+	+	-	Actin-, S100-, CD10-, STAT6-, ALK-	ND
15	spindle	Absent	3	+	ND	+	-	SMA-, S100-	ND
16	epithelioid	Present	53	+	ND	+	-	AE1/AE3-, SMA-. S100-, Melan A-	ND
17	epithelioid and spindle	Present	33	+	ND	+	ND	ND	ND
18	epithelioid and spindle	Absent	2	+	+	ND	-	AE1/AE3-, Cam5.2-, CD10-, SF1-, Cyclin D1-, ER-, PR+ (weak), SDHB +	FGFR3 p.G145S
19	epithelioid	Absent	27	+	ND	ND	+ (weak)	AE1/AE3-, SMA-, S100-	ND
20	spindle	Present	25	+	+	ND	-	AE1/AE3-, HMB45-, Melan A-, Cathepsin K+	ND

HPF: High power field; IHC: Immunohistochemistry; MF: Mitotic figure; ND: Not done.

## References

[1] Søreide K., Sandvik O.M., Søreide J.A., Giljaca V., Jureckova A., Bulusu V.R. (2016). Global epidemiology of gastrointestinal stromal tumours (GIST): A systematic review of population-based cohort studies. Cancer Epidemiol..

[2] Nilsson B., Bümming P., Meis-Kindblom J.M., Odén A., Dortok A., Gustavsson B., Sablinska K., Kindblom L.G. (2005). Gastrointestinal stromal tumors: The incidence, prevalence, clinical course, and prognostication in the preimatinib mesylate era--a population-based study in western Sweden. Cancer.

[3] Rossi S., Miceli R., Messerini L., Bearzi I., Mazzoleni G., Capella C., Arrigoni G., Sonzogni A., Sidoni A., Toffolatti L. (2011). Natural history of imatinib-naive GISTs: A retrospective analysis of 929 cases with long-term follow-up and development of a survival nomogram based on mitotic index and size as continuous variables. Am. J. Surg. Pathol..

[4] Kindblom L.G., Remotti H.E., Aldenborg F., Meis-Kindblom J.M. (1998). Gastrointestinal pacemaker cell tumor (GIPACT): Gastrointestinal stromal tumors show phenotypic characteristics of the interstitial cells of Cajal. Am. J. Pathol..

[5] Miettinen M., Lasota J. (2006). Gastrointestinal stromal tumors: Review on morphology, molecular pathology, prognosis, and differential diagnosis. Arch. Pathol. Lab. Med..

[6] Miettinen M., Lasota J. (2006). Gastrointestinal stromal tumors: Pathology and prognosis at different sites. Semin. Diagn. Pathol..

[7] Novelli M., Rossi S., Rodriguez-Justo M., Taniere P., Seddon B., Toffolatti L., Sartor C., Hogendoorn P.C., Sciot R., Van Glabbeke M. (2010). DOG1 and CD117 are the antibodies of choice in the diagnosis of gastrointestinal stromal tumours. Histopathology.

[8] Joensuu H., Rutkowski P., Nishida T., Steigen S.E., Brabec P., Plank L., Nilsson B., Braconi C., Bordoni A., Magnusson M.K. (2015). KIT and PDGFRA mutations and the risk of GI stromal tumor recurrence. J. Clin. Oncol..

[9] Emile J.F., Brahimi S., Coindre J.M., Bringuier P.P., Monges G., Samb P., Dosucet L., Hostein I., Landi B., Buisine M.P. (2012). Frequencies of KIT and PDGFRA mutations in the MolecGIST prospective population-based study differ from those of advanced GISTs. Med. Oncol..

[10] Corless C.L., Schroeder A., Griffith D., Town A., McGreevey L., Harrell P., Shiraga S., Bainbridge T., Morich J., Heinrich M.C. (2005). PDGFRA mutations in gastrointestinal stromal tumors: Frequency, spectrum and in vitro sensitivity to imatinib. J. Clin. Oncol..

[11] Daniels M., Lurkin I., Pauli R., Erbstösser E., Hildebrandt U., Hellwig K., Zschille U., Lüders P., Krüger G., Knolle J. (2011). Spectrum of KIT/PDGFRA/BRAF mutations and Phosphatidylinositol-3-Kinase pathway gene alterations in gastrointestinal stromal tumors (GIST). Cancer Lett..

[12] Gasparotto D., Rossi S., Polano M., Tamborini E., Lorenzetto E., Sbaraglia M., Mondello A., Massani M., Lamon S., Bracci R. (2017). Quadruple-Negative GIST Is a Sentinel for Unrecognized Neurofibromatosis Type 1 Syndrome. Clin. Cancer Res..

[13] Boikos S.A., Pappo A.S., Killian J.K., LaQuaglia M.P., Weldon C.B., George S., Trent J.C., von Mehren M., Wright J.A., Schiffman J.D. (2016). Molecular Subtypes of KIT/PDGFRA Wild-Type Gastrointestinal Stromal Tumors: A Report from the National Institutes of Health Gastrointestinal Stromal Tumor Clinic. JAMA Oncol..

[14] Belinsky M.G., Rink L., Cai K.Q., Capuzzi S.J., Hoang Y., Chien J., Godwin A.K., von Mehren M. (2015). Somatic loss of function mutations in neurofibromin 1 and MYC associated factor X genes identified by exome-wide sequencing in a wild-type GIST case. BMC Cancer.

[15] Menge F., Jakob J., Kasper B., Smakic A., Gaiser T., Hohenberger P. (2018). Clinical Presentation of Gastrointestinal Stromal Tumors. Visc. Med..

[16] Ijeri S.K., Rathod P.S., Kundargi R., Pallavi V.R., Shobha K., Shankaranand, Vijay C.R., Uma Devi K., Bafna U.D. (2016). Gastrointestinal Stromal Tumor Mimicking as Ovarian Tumor in Gynaecologic Oncology. Indian J. Surg. Oncol..

[17] Yamaguchi T., Kinoshita J., Saito H., Shimada M., Terai S., Moriyama H., Okamoto K., Nakamura K., Tajima H., Ninomiya I. (2021). Gastrointestinal stromal tumor metastasis to the ovary: A case report. SAGE Open Med. Case Rep..

[18] Hwang S.Y., Choi C.I., Cho H.J., Kim D.H., Hong S.B., Choi K.U., Suh D.S. (2020). A ruptured jejunal gastrointestinal stromal tumor with hemoperitoneum mimicking ovarian carcinoma. Int. J. Clin. Exp. Pathol..

[19] Irving J.A., Lerwill M.F., Young R.H. (2005). Gastrointestinal stromal tumors metastatic to the ovary: A report of five cases. Am. J. Surg. Pathol..

[20] Angioli R., Battista C., Muzii L., Terracina G.M., Cafà E.V., Sereni M.I., Montera R., Plotti F., Rabitti C., Panici P.B. (2009). A gastrointestinal stromal tumor presenting as a pelvic mass: A case report. Oncol. Rep..

[21] De Leo A., Nannini M., Dondi G., Santini D., Urbini M., Gruppioni E., De Iaco P., Perrone A.M., Pantaleo M.A. (2018). Unusual bilateral ovarian metastases from ileal gastrointestinal stromal tumor (GIST): A case report. BMC Cancer.

[22] Rahma D.Y., Atmaja M.H.S. (2022). Gastrointestinal stromal tumor as mimicking gynecological mass finding on CT scan imaging: A case report. Int. J. Surg. Case Rep..

[23] Kobayashi M., Otsuki Y., Kobayashi H., Suzuki T., Nakayama S., Adachi H. (2022). Gastrointestinal Stromal Tumor with Repeated Multiple Cerebral Infarction Mimicking Ovarian Cancer with Trousseau’s Syndrome. Case Rep. Obstet. Gynecol..

[24] Pelz A.F., Agaimy A., Daniels M., Evert M., Schulz H.U., Lüders P., Müller G., Lasota J., Röpke A., Wieacker P. (2011). Gastrointestinal stromal tumor presenting as a rectovaginal mass. Clinicopathologic and molecular-genetic characterization of a rare tumor with a literature review. Hum. Pathol..

[25] Lam M.M., Corless C.L., Goldblum J.R., Heinrich M.C., Downs-Kelly E., Rubin B.P. (2006). Extragastrointestinal stromal tumors presenting as vulvovaginal/rectovaginal septal masses: A diagnostic pitfall. Int. J. Gynecol. Pathol..

[26] Xing D., Zheng G., Schoolmeester J.K., Li Z., Pallavajjala A., Haley L., Conner M.G., Vang R., Hung C.F., Wu T.C. (2018). Next-generation Sequencing Reveals Recurrent Somatic Mutations in Small Cell Neuroendocrine Carcinoma of the Uterine Cervix. Am. J. Surg. Pathol..

[27] Xing D., Schoolmeester J.K., Ren Z., Isacson C., Ronnett B.M. (2016). Lower Female Genital Tract Tumors With Adenoid Cystic Differentiation: P16 Expression and High-risk HPV Detection. Am. J. Surg. Pathol..

[28] Xing D., Zheng G., Pallavajjala A., Schoolmeester J.K., Liu Y., Haley L., Hu Y., Liu L., Logan L., Lin Y. (2019). Lineage-Specific Alterations in Gynecologic Neoplasms with Choriocarcinomatous Differentiation: Implications for Origin and Therapeutics. Clin. Cancer Res..

[29] Shrestha S., Shrestha B.M., Kharel S., Rijal Y., Joshi J.P., Tiwari S.B., Sah J.K., Ghimire B. (2021). Jejunal GIST masquerading as an ovarian mass: A case report. Int. J. Surg. Case Rep..

[30] Turner L.M., Jeans P., Robson S. (2021). A pedunculated small bowel gastrointestinal stromal tumour (GIST) masquerading as an ovarian tumour. J. Surg. Case Rep..

[31] Kurman R.J., Carcangiu M.L., Herrington C.S., Young R.H. (2014). WHO Classification of Tumors of Female Reproductive Organs.

[32] Fletcher C.D., Berman J.J., Corless C., Gorstein F., Lasota J., Longley B.J., Miettinen M., O’Leary T.J., Remotti H., Rubin B.P. (2002). Diagnosis of gastrointestinal stromal tumors: A consensus approach. Hum. Pathol..

[33] Sanneh A., Murdock T., Wethington S.L., Fader A.N., Xing D., Beavis A.L. (2020). Low-Grade Endometrial Stromal Sarcoma Diagnosed 8 Years After Hysterectomy With Morcellation. Obstet. Gynecol..

[34] Schoolmeester J.K., Howitt B.E., Hirsch M.S., Dal Cin P., Quade B.J., Nucci M.R. (2014). Perivascular epithelioid cell neoplasm (PEComa) of the gynecologic tract: Clinicopathologic and immunohistochemical characterization of 16 cases. Am. J. Surg. Pathol..

[35] Dahoud W., Handler J., Parimi V., Meyer C.F., Wethington S.L., Eshleman J.R., Vang R., Ronnett B.M., Xing D. (2021). Adult Granulosa Cell Tumor with Sarcomatous Transformation: A Case Study With Emphasis on Molecular Alterations. Int. J. Gynecol. Pathol..

[36] Merten L., Agaimy A., Moskalev E.A., Giedl J., Kayser C., Geddert H., Schaefer I.M., Cameron S., Werner M., Ströbel P. (2016). Inactivating Mutations of RB1 and TP53 Correlate With Sarcomatous Histomorphology and Metastasis/Recurrence in Gastrointestinal Stromal Tumors. Am. J. Clin. Pathol..

[37] Heinrich M.C., Patterson J., Beadling C., Wang Y., Debiec-Rychter M., Dewaele B., Corless C.L., Duensing A., Raut C.P., Rubin B. (2019). Genomic aberrations in cell cycle genes predict progression of KIT-mutant gastrointestinal stromal tumors (GISTs). Clin. Sarcoma Res..

[38] Astolfi A., Pantaleo M.A., Indio V., Urbini M., Nannini M. (2020). The Emerging Role of the FGF/FGFR Pathway in Gastrointestinal Stromal Tumor. Int. J. Mol. Sci..

[39] Mavroeidis L., Metaxa-Mariatou V., Papoudou-Bai A., Lampraki A.M., Kostadima L., Tsinokou I., Zarkavelis G., Papadaki A., Petrakis D., Gκoura S. (2018). Comprehensive molecular screening by next generation sequencing reveals a distinctive mutational profile of KIT/PDGFRA genes and novel genomic alterations: Results from a 20-year cohort of patients with GIST from north-western Greece. ESMO Open..

[40] Park J., Yoo H.M., Sul H.J., Shin S., Lee S.W., Kim J.G. (2020). Genetic Characterization of Molecular Targets in Korean Patients with Gastrointestinal Stromal Tumors. J. Gastric Cancer.

[41] Javidi-Sharifi N., Traer E., Martinez J., Gupta A., Taguchi T., Dunlap J., Heinrich M.C., Corless C.L., Rubin B.P., Druker B.J. (2015). Crosstalk between KIT and FGFR3 Promotes Gastrointestinal Stromal Tumor Cell Growth and Drug Resistance. Cancer Res..

